# Mechanoluminescent Aluminum Nitride Crystal for Super‐Sensitive Optical Manometry, Thermometry and Force Sensing

**DOI:** 10.1002/adma.202511943

**Published:** 2025-09-09

**Authors:** Teng Zheng, Przemysław Woźny, Kevin Soler‐Carracedo, Dongxue Han, Jie Wang, Liang Peng, Wenliang Li, Dengfeng Peng, Honglei Wu, Jan Moszczyński, Sebastian Mahlik, Marcin Runowski

**Affiliations:** ^1^ School of Information and Electrical Engineering Hangzhou City University Hangzhou 310015 China; ^2^ Faculty of Chemistry Adam Mickiewicz University Uniwersytetu Poznańskiego 8 Poznań 61–614 Poland; ^3^ Institute of Experimental Physics Faculty of Mathematics Physics and Informatics University of Gdansk Wita Stwosza 57 Gdansk 80–308 Poland; ^4^ Shenzhen Key Laboratory of Intelligent Optical Measurement and Detection Shenzhen University Shenzhen 518060 China; ^5^ Key Laboratory of Optoelectronic Devices and Systems of Ministry of Education and Guangdong Province College of Physics and Optoelectronic Engineering Shenzhen University Shenzhen 518060 China; ^6^ State Key Laboratory of Radio Frequency Heterogeneous Integration Shenzhen University Shenzhen 518060 China

**Keywords:** aluminium nitride, luminescent temperature sensor, mechanoluminescence, optical manometry, ultra‐wide bandgap semiconductor

## Abstract

AlN is a core material widely used as a substrate and heat sink in various electronic and optoelectronic devices. Introducing luminescent properties into intrinsic AIN opens new opportunities for next‐generation intelligent sensors, self‐powered displays, and wearable electronics. In this study, the first evidence is presented of AlN crystals exhibiting satisfactory mechanoluminescence (ML), photoluminescence (PL), and afterglow performance, demonstrating their potential as novel multifunctional optical sensors. A series of undoped AlN crystals (ranging from µm to mm scale) is successfully synthesized on tungsten substrates via physical vapor transport. A multimodal optical sensing platform is developed, showing afterglow and PL for temperature and pressure sensing, and ML for force detection. Despite minimal structural deformation under extreme conditions, attributed to the high bulk modulus of AIN, the optical sensors, driven by intrinsic defect‐related emissions, exhibited excellent sensitivity to temperature and pressure. Notably, significant and previously unreported differences in the PL and ML spectra are observed in response to light and mechanical stimuli, respectively. These spectral variations are attributed to the activation of distinct defect states during PL and ML processes. This proof‐of‐concept study represents a significant step forward in the development of optical sensing technologies for extreme environments and force detection applications.

## Graphical abstract

This study reports the first discovery of intrinsic mechanoluminescence in undoped AlN crystals, accompanied by pronounced photoluminescence and persistent luminescence. It further demonstrates a defect‐emission–driven multifunctional optical sensing platform for force, pressure, and temperature detection, achieving exceptional sensitivities.

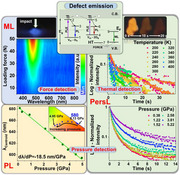

## 1 Introduction

III‐nitride semiconductor materials, including AlN, GaN, and InN, play a pivotal role in the ongoing revolution of modern electronics and optoelectronics technology.^[^ ^1^, ^2^, ^3^ ^]^ Among them, AlN stands out as a state‐of‐the‐art ultra‐wide bandgap semiconductor, characterized by a wide bandgap (≈6.2 eV), high electrical resistivity (10^9^–10^−11^ Ω·m), excellent thermal conductivity (170–220 W m·K^−1^), and outstanding thermal and chemical stability.^[^ ^4^, ^5^ ^]^ Beyond its established applications in electronics and semiconductor devices, AIN is also utilized in blue‐violet light‐emitting diodes, short‐wavelength lasers, and ultraviolet (UV) detectors.^[^ ^5^, ^6^ ^]^ The utility of AIN arises largely from the presence of various intrinsic and extrinsic defects, whose energy levels are localized within its wide bandgap. These defects serve as luminescent centers, enabling efficient light emission across a broad spectral range, from UV to visible wavelengths.^[^ ^7^, ^8^ ^]^ AlN typically contains native defects, including aluminium (V_Al_), nitrogen (V_N_), and oxygen‐related (V_Al_‐O_N_) vacancies and interstitials.^[^ ^9^, ^10^ ^]^ The optical emissions resulting from these complex defects can be modulated by external stimuli such as temperature, pressure, and mechanical force. Such environment‐responsive luminescence is of significant interest for advancing emerging technologies, including 5G communications, electric vehicles, high‐speed rail systems, smart grids, wearable electronics, robotics, and high‐pressure sensing.^[^ ^11^ ^]^ Therefore, a deeper understanding of the luminescence properties and the underlying defect mechanisms in AIN under extreme conditions is needed.

Mechanoluminescence (ML) refers to the emission of visible, UV, or near‐infrared radiation from a material when subjected to mechanical stress. This phenomenon arises from mechanical deformation or internal stress that can be initiated by mechanical actions, including compression, bending, stretching, static loading, pressure pulses, impact deformation, cleavage, cutting, friction, grinding, vibration, scratching, and even mechanical waves. Such a unique transduction mechanism from force to photon largely boosts its stress‐sensing applications.^[^ ^12^, ^13^ ^]^ Great efforts have been devoted to developing ML‐based stress sensors, which show great potential in advanced artificial skin, human‐computer interaction, and robotics.^[^ ^13^, ^14^, ^15^, ^16^, ^17^, ^18^ ^]^ Notably, recent research demonstrates that ML‐based materials can detect forces with high spatial resolution—even when those forces occur inside a microscopic living worm.^[^ ^19^ ^]^ Currently, most ML‐active materials are based on inorganic host matrices doped with rare‐earth or *d*‐block transition metal ions.^[^ ^20^ ^]^ However, undoped ML materials offer several distinct advantages, such as the elimination of dopant‐induced disruptions, enhanced chemical purity, and the reduction of quenching centers. These benefits make undoped materials ideal for studying intrinsic ML mechanisms. Therefore, the development of high‐performance undoped ML materials is essential to meet the growing demand for advanced sensing applications. Moreover, investigating ML in undoped materials can provide deeper insights into the role of intrinsic defects and charge traps in light emission, thereby advancing the fundamental understanding and theoretical design of next‐generation ML materials.

To date, only a few undoped host materials have been reported to exhibit ML. Zhang et al. synthesized undoped CaZnOS under an argon atmosphere, introducing various types of oxygen vacancies, and demonstrated oxygen vacancy‐mediated, multimechanoresponsive green ML emission.^[^ ^9^ ^]^ More recently, a combination of quantum mechanical calculations and experimental analysis revealed the evolution of crystal structure under mechanical stress in undoped CaLaAl_3_O_7_, including the formation of additional oxygen‐ and calcium‐related defects.^[^ ^21^ ^]^ These defects are believed to be responsible for the distinct spectral differences observed between photoluminescence (PL) and ML in the material. Although these studies have provided valuable insights into ML mechanisms in undoped hosts, particularly from the viewpoint of defect and trap dynamics, the ML performance of these materials still lags behind that of conventional doped systems. In particular, undoped hosts often suffer from limited ML intensity and transient emitting behavior, which significantly restricts their practical applicability. According to general ML theory, a high energy transfer efficiency between localized energy levels (LELs) and trap states is essential for achieving strong ML emission in undoped materials.^[^ ^22^, ^23^ ^]^

Pressure and temperature, as two fundamental thermodynamic variables, significantly influence not only everyday phenomena but also the physicochemical properties of materials. Under extreme pressure and temperature conditions, materials can undergo phase transitions or form entirely new phases with unique properties. For example, nearly half of the more than 50 known superconducting materials have been synthesized under high‐pressure conditions.^[^ ^24^ ^]^ High pressure can also induce luminescence in otherwise non‐emissive materials^[^ ^25^ ^]^ or facilitate the formation of novel substances and crystal structures.^[^ ^26^, ^27^, ^28^ ^]^ As such, accurate and rapid pressure measurement is critical for exploring and utilizing these phenomena. Due to their optical transparency in the visible range, diamonds and other gemstones serve as ideal media for optical (luminescent) pressure calibrants. For example, ruby (Al_2_O_3_: Cr^3+^) and samarium‐doped materials are commonly used for precise pressure measurements.^[^ ^29^, ^30^ ^]^ In contrast, luminescent thermometers typically rely on phosphors and organic dyes doped with lanthanide ions, which exhibit characteristic 4*f*‐4*f* transitions and ladder‐like energy levels. However, conventional luminescence intensity ratio (LIR) thermometers based on thermally‐coupled levels (TCLs) of lanthanide ions, such as Er^3+^, Tm^3+^, and Nd^3+^, are inherently limited in sensitivity due to the fixed and narrow energy gaps between TCLs (200 ≤ ΔE ≤ 2000 cm^−1^).^[^ ^31^ ^]^ A promising strategy to overcome these limitations involves the use of optical band‐shift sensors based on defect‐related emissions. Defect states can serve as efficient trapping and recombination centers for charge carriers, and their recombination processes are highly sensitive to temperature. Additionally, the optical behavior of such materials is intrinsically related to the local energy states of the defect sites.

Herein, we demonstrate a multimodal optical sensor based on defect‐related emissions in undoped AlN, capable of temperature and pressure sensing via PL and force detection via ML. A series of piezoelectric AlN crystals, synthesized under different synthesis conditions, was systematically optimized to enhance PL and ML performance for optical sensing applications. Notably, the synthesized AlN crystals exhibited significant and rarely reported differences between their PL and ML spectra under light and mechanical excitation, respectively. These spectral differences are attributed to the activation of distinct defect states involved in the generation of PL and ML, indicating the critical role of defect‐specific emission pathways in multimodal sensing.

## 2. Results and Discussion

### 1 Basic Properties at Ambient Condition

**Figure 1:**
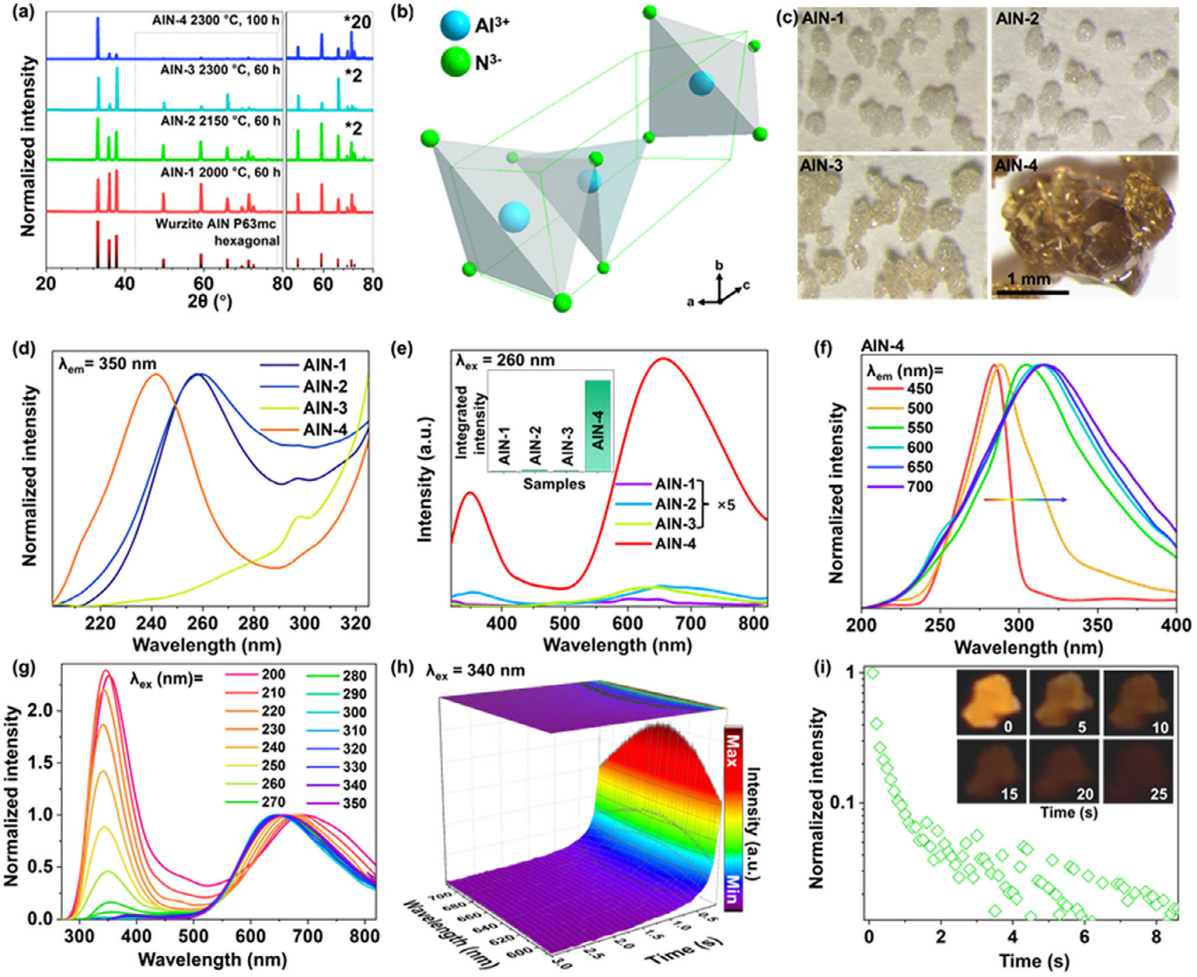
Structural and PL properties of AIN samples under ambient conditions. a) Powder XRD patterns of AIN samples synthesized under different sintering conditions, i.e., the AlN‐1, AlN‐2, AlN‐3, and AlN‐4; The right part of the figure shows the magnified region of the patterns with varying multiplication factors. b) 3D graphical representation of the hexagonal wurtzite AlN crystal structure. c) Optical microscopy images of the synthesized samples. d) Normalized PL excitation spectra recorded at λ_em_ = 350 nm. e) PL emission spectra of the samples recorded at λ_ex_ = 260 nm; the inset shows the integrated emission intensities of the samples. f) Normalized PL excitation spectra of the AlN‐4 sample as a function of λ_em_. g) PL emission spectra of the AlN‐4 sample as a function of λ_ex_, normalized to the maximum intensity of the red emission band. h) Time‐resolved emission spectra of AlN‐4 measured at λ_ex_ = 340 nm at room temperature. i) Corresponding luminescence decay curve; the inset shows a series of luminescence photographs captured at different time intervals following removal of the excitation source.

The synthesis details are provided in Supporting Information. AlN samples were synthesized at different high‐temperature sintering conditions, *i.e*., 2000 °C for 60 h (designated AlN‐1), 2150 °C for 60 h (AlN‐2), 2300 °C for 60 h (AlN‐3), and 2300 °C for 100 h (AlN‐4). The powder X‐ray diffraction (XRD) patterns of AlN‐1 through AlN‐4 are shown in **Figure** **1**a. All diffraction peaks match the reference data from the Crystallography Open Database (COD), card no. 96‐101‐0515, confirming the formation of hexagonal wurtzite AlN (space group: P6_3_mc, no. 186). Elemental analysis via energy‐dispersive X‐ray spectroscopy (EDX) for the optimized AlN‐4 sample (Figure S1, Supporting Information) confirms the presence of aluminum and nitrogen, consistent with the AIN composition. A 3D representation of the wurtzite‐type AIN unit cell is provided in Figure 1b. Due to the large crystallite size, the sample morphologies are examined through microscopy (Figure 1c), revealing that AlN‐1, AIN‐2, and AlN‐3 consist of irregular, micron‐sized particles (200–600 µm) regardless of synthesis conditions. In contrast, AlN‐4 exhibits a distinct morphology, characterized by a single‐crystal nature, irregular shape, and significantly larger particle size (1–2 mm in diameter), 5–10 times than that of the other samples. Notably, initial tests revealed that AlN‐4 displayed significantly stronger ML and PL compared to the other synthesized AlN samples. Consequently, AIN‐4 was selected for detailed characterization and analysis.

The diffuse reflectance spectra of the synthesized AlN materials are shown in Figure S2 (Supporting Information). Among the samples, AlN‐4 exhibited significantly lower reflectance, corresponding to higher optical absorption, across the spectral range of 240–1700 nm. Figure 1d displays the normalized PL excitation spectra of the AlN samples recorded at an emission wavelength (λ_em_) of 350 nm. All samples exhibited a relatively broad excitation band between 200 and 325 nm, with centroid positions varying across the series: 240 nm for AlN‐4, 260 nm for AlN‐1 and AlN‐2, and 325 nm for AlN‐3. As shown in Figure 1e, the PL emission intensities of AlN‐1, AlN‐2, and AlN‐3 (excited at λ_ex_ = 260 nm) are considerably lower than that of AlN‐4; therefore, their spectra are magnified by a factor of 5 for comparison. In addition to intensity differences, the emission band shapes varied significantly among the samples, with distinct peak positions despite being excited at the same wavelength. These spectral variations are likely attributed to the differences in high‐temperature sintering conditions during synthesis, which influence the formation of defect levels within the wide bandgap. These variations in defect depth and energy distribution result in different emitting centers across the UV to visible spectral range, ultimately affecting the emission intensity, spectral shape, and peak positions.

As shown in Figure 1f, tuning λ_em_ from 450 to 700 nm for the representative AlN‐4 sample, which exhibits the highest luminescence performance, results in a noticeable redshift and broadening of the excitation band, indicating a unidirectional spectral expansion. Conversely, when λ_ex_ was varied from 200 to 350 nm (Figure 1g), substantial changes were observed in the relative intensities of the emission bands located in the UV and red regions. Specifically, the intensity of the high‐energy emission band centered ≈350 nm (UV) increased significantly relative to the low‐energy red emission band as the excitation energy increased (i.e., with decreasing λ_ex_). Notably, the emission bands exhibited broad, asymmetrical peak shapes, suggesting contributions from multiple deep energy levels associated with crystal defect states. These observations are consistent with prior reports in the literature.^[^ ^32^, ^33^, ^34^ ^]^ Furthermore, as λ_ex_ increased, the red emission band exhibited a significant blueshift, likely resulting from variations in the relative contributions of multiple emission components within the band. The UV‐blue emission band is commonly attributed to intrinsic crystal defects, such as V_N_ vacancies, (O_N_)^−^, or aluminum interstitials formed during high‐temperature synthesis. Oxygen, in particular, is a known natural dopant in AlN.^[^ ^32^, ^35^ ^]^ Importantly, the presence of such defects or impurities can lead to significant defect states or impurity level excitations without exceeding the fundamental bandgap. The spectral position of the UV‐blue emission band in this study aligns well with previously reported data,^[^ ^36^, ^37^ ^]^ with minor deviations likely arising from differences in oxygen concentration within the AlN lattice.

Additionally, the potential contribution of unintentional Mn impurities, specifically, divalent Mn^2+^ ions substituting Al^3+^ in the host lattice, is considered as a possible origin of the red emission band, given that Mn^2+^ is known to produce characteristic red luminescence. To investigate this possibility, the PL emission spectrum of the AIN‐4 sample is measured at low temperature (77 K), as shown in Figure S3 (Supporting Information). The absence of the characteristic phonon structure (i.e., superimposed narrow peaks) typically associated with Mn^2+^‐related luminescence, along with a significant spectral shift relative to standard Mn^2+^ emission, clearly indicates that Mn ions are not present in the material. This finding supports the assignment of the red emission band to structural defects within the AlN crystal lattice.^[^ ^38^, ^39^ ^]^ To further explore the behavior of the two distinct emission bands, PL spectra are recorded as a function of λ_ex_ ranging from 200 to 350 nm, as shown in Figure S4a–c (Supporting Information). Initially, as λ_ex_ increased from 200 to 240 nm (Figure S4a, Supporting Information), the intensities of both the UV‐blue and red emission bands increased. In the intermediate range of 250–290 nm (Figure S4b, Supporting Information), the intensity of the UV‐blue emission gradually decreased, while the red emission band continued to increase. Finally, at higher λ_ex_ (300–350 nm; Figure S4c, Supporting Information), the UV‐blue emission became negligible, its intensity approaching the noise level, while the red emission band began to decrease in intensity. Time‐resolved emission spectra and corresponding luminescence decay curves are presented in Figure 1h,i. These results reveal that the AlN‐4 sample exhibits excellent persistent luminescence, with a notably long excited‐state lifetime of ≈0.59 s. As shown in the inset of Figure 1i, luminescence photographs of the sample demonstrate a distinct red‐orange afterglow, with intense, visually detectable emission persisting for up to 25 s after the excitation source is removed.

### 2 ML Properties

**Figure 2:**
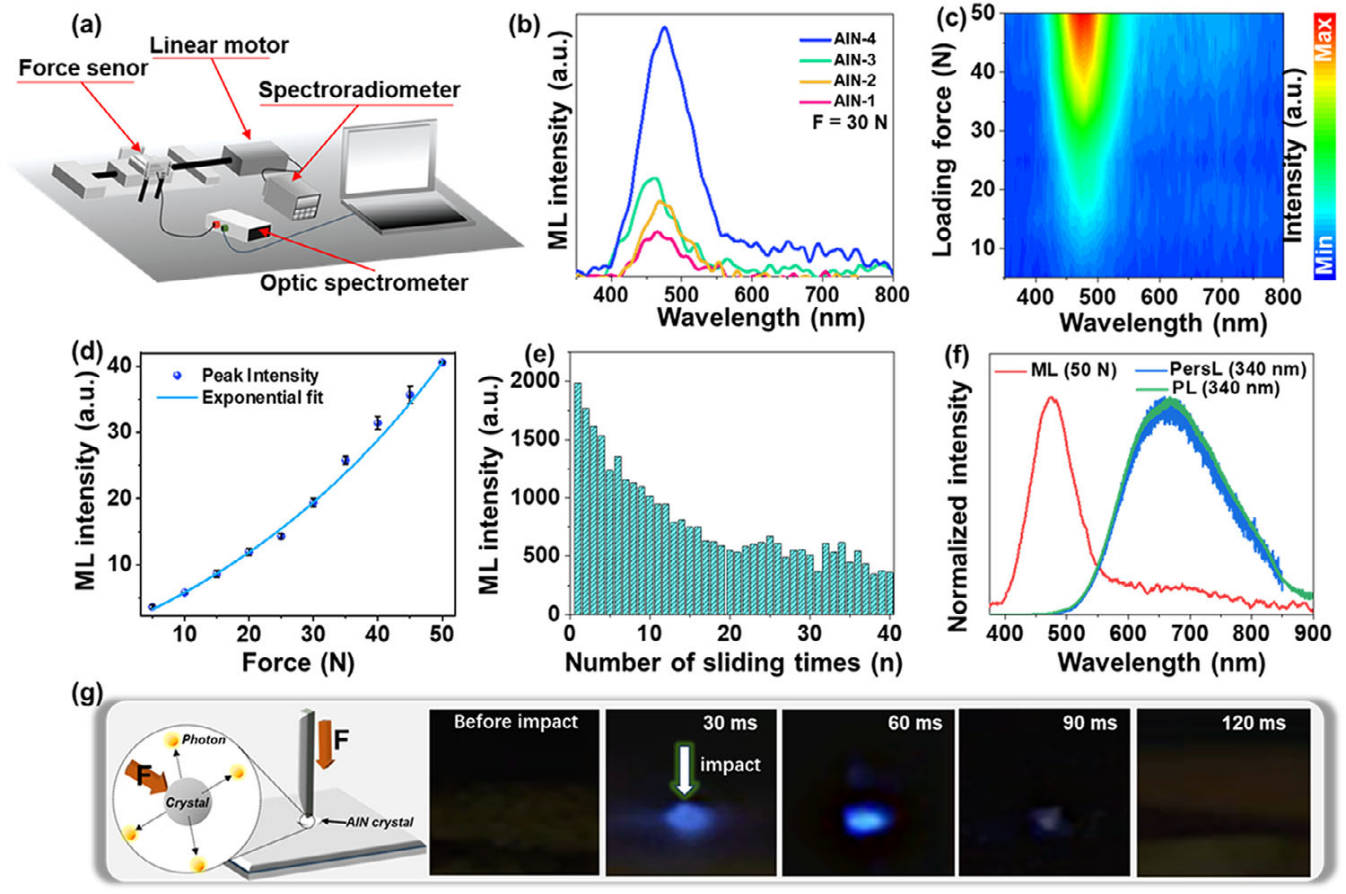
ML properties of AIN materials. a) Schematic of the experimental setup used for ML measurements on the AlN samples. b) Comparison of ML emission spectra for different synthesized samples under a mechanical loading force of ≈30 N. c) ML spectra of the AIN‐4 sample recorded under varying loading force. d) Dependence of total ML intensity on applied force for the AIN‐4 sample, with experimental data fitted to an exponential function. e) Integrated ML signal generated by 40 successive movements of a glass rod across the AIN‐4 sample, with a constant applied force of 10 N. f) Normalized emission spectra of ML (50 N applied force), PersL, and PL at (λ_ex_ = 340 nm) for the AlN‐4 sample. g) Schematic and digital photographs of AIN‐4 crystals exhibiting bright blue ML under dynamic mechanical impact (≈50 N), captured in darkness.

A simplified experimental setup for investigating the ML properties of the selected AlN‐4 sample is shown in **Figure** **2**a, with additional experimental details provided in the SI. Before ML measurements, the sample was excited using a 280‐nm UV diode for 10 min and subsequently kept in the dark until the persistent luminescence signal had sufficiently decayed. As shown in Figure 2b, ML spectra are recorded under a mechanical load of 30 N applied uniformly across all samples. The ML emission bands were centered ≈475 nm and extended across the 400–800 nm range. Notably, the ML intensity increased with higher sintering temperatures and longer synthesis times. Figure 2c presents the ML spectra of the optimized AlN‐4 sample under increasing impact forces, and Figure 2d illustrates the corresponding dependence of total ML intensity on the applied force. The total ML intensity exhibited a strong exponential relationship with the applied force, with a fitting correlation coefficient R^2^>0.999. The following exponential function was applied:

(1) $$I=A_{1}\times \exp\left(-F/B_{1}\right)+A_{2}\times \exp\left(-F/B_{2}\right)+y_{0}$$

where *I* represents the ML intensity, *F* is the applied force, and *A_1_*, *A_2_*, *B_1_*, *B_2_*, and *y_0_* are fitting constants, as shown in SI. To further investigate the correlation between ML intensity and applied force, the relative intensity change was normalized, with the intensity at 50 N defined as 100%. The relative intensity (I/I_50_) and corresponding sensitivity are shown in Figure S5a,b (Supporting Information). The maximum sensitivity, reaching 3.21%/N at an applied force of 50 N, demonstrates the excellent sensitivity of the developed force sensor based on ML. In the elastic limit of the material, a fundamental principle of stress sensing is the conversion of applied mechanical stress into a measurable output signal, enabling a predictable monotonic correlation between force magnitude and signal intensity.^[^ ^12^ ^]^ The observed nonlinear, exponential behavior of ML intensity as a function of force likely arises from multiple fundamental mechanisms. These may include: i) the presence of various defect states and impurity levels, from which charge carriers are released and recombined at varying force levels (corresponding to different trap depths and energies), and/or ii) the simultaneous contribution of the triboelectric effects at lower forces and the dominance of the piezoelectric effect at higher force regimes.^[^ ^40^, ^41^ ^]^ A notable advantage of AlN is its ability to exhibit self‐recovering ML behavior, emitting light under repeated mechanical stimuli without requiring re‐excitation by UV light. Figure 2e illustrates the sustainability and repeatability of the ML signal under repeated mechanical stimuli. Although the ML signal decreased gradually as the number of loading cycles and longer sliding durations, it retained relatively stable performance throughout the test. Additionally, Figure S6 (Supporting Information) presents ML spectra recorded under a 30 N impact force at 1, 24, and 48 h after initial UV excitation. The integrated ML intensity decreased to ≈73% of its original value after 24 h and remained nearly constant from 24 to 48 h, indicating good stability of the trapped charge carriers over time.

To investigate the mechanisms underlying the ML, PL, and persistent luminescence (PersL) phenomena in the synthesized AlN material, the normalized ML, PL, and PersL emission spectra of the AlN‐4 sample are compared in Figure 2f. Figure 1g shows the digital photographs of the AlN‐4 crystals during ML, captured in darkness under dynamic mechanical stimulation (≈50 N impact by a metal rod). These images visually demonstrate the blue luminescence response of the crystals during impact, further supporting the proposed ML mechanism. Videos demonstrating the ML behavior of AlN‐4 crystals under impact stimulation are provided in Video S1 (Supporting Information).

**Figure 3:**
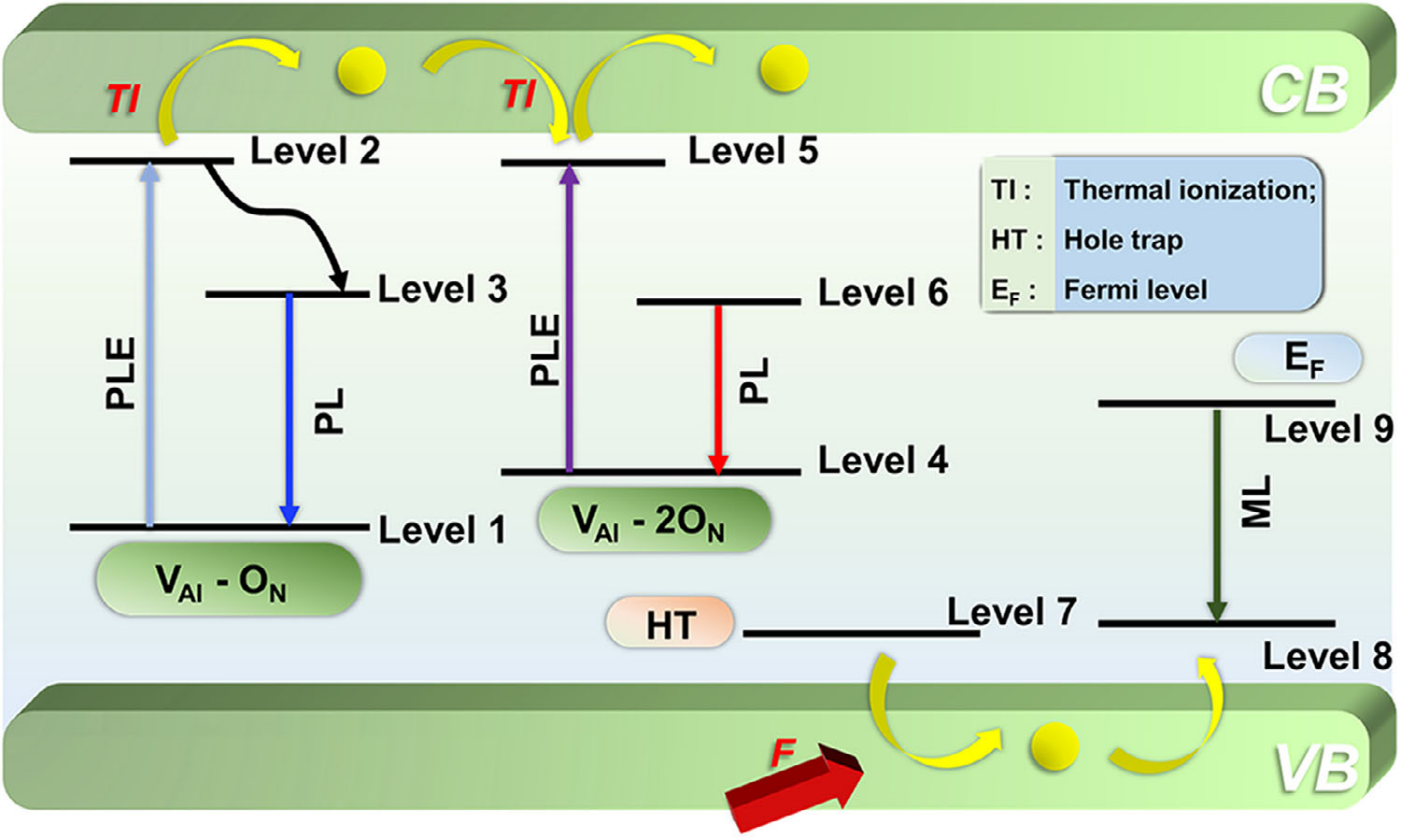
Proposed defect‐related energy‐level diagram illustrating the mechanisms underlying ML, PL, and PersL phenomena in AIN, including possible excitation and radiative recombination pathways.

The optical properties are interpreted based on a proposed defect‐related energy‐level diagram in **Figure** **3**. Both the PL and PersL (afterglow) spectra exhibited nearly identical spectral profiles, broad emission bands centered ≈650 nm in the red region, suggesting that similar defect‐related recombination pathways were responsible for both phenomena. In contrast, the ML spectrum was distinctly different, exhibiting a pronounced blue emission centered at ≈475 nm, indicative of an alternative recombination mechanism specific to mechanical excitation. The blue emission in AlN observed under mechanical stimulation is attributed to the presence of complex crystal defects, particularly those involving V_Al_ and oxygen substituting for nitrogen (O_N_).^[^ ^42^, ^43^ ^]^ In this model, the ground state of the V_Al_–O_N_ defect complex (level 1) lies ≈2.25 eV above the valence band, while its excited state (level 2) is estimated to lie ≈0.5 eV below the CB.^[^ ^44^ ^]^ Upon UV excitation, the defect center transitions to its excited state (V_Al_–O_N_)^*^ (level 2). When this complex is close to a neighboring O_N_ defect (level 3), energy transfer leads to radiative recombination between V_Al_–O_N_^+^ and O_N_^–^, producing blue luminescence. However, if the V_Al_–O_N_ complex is spatially isolated from other defects, level 2 acts as an electron trap, and it is responsible for the afterglow luminescence.^[^ ^44^ ^]^ Because of its proximity to the CB, thermal ionization (TI) can release trapped electrons into the CB. These electrons may later be trapped and subsequently recombine, resulting in delayed radiative emission, which is observed as persistent luminescence.^[^ ^45^, ^46^, ^47^ ^]^ The red emission band centered near 650 nm is also attributed to defect complexes involving V_Al_ and substitutional O_N_, particularly V_Al_–2O_N_ centers. In Figure 3, state 4 represents the ground state of this complex, and state 5 denotes its excited state. This excited state can be directly populated by PL excitation (PLE) and electrons from this level may either (i) undergo thermal ionization into the CB, followed by recapture and delayed radiative emission or (ii) be transferred to a nearby defect level (state 6), with subsequent radiative recombination from state 6 to state 4 producing red emission near 580–600 nm. The green luminescence band observed exclusively during ML, centered at ≈475 nm, is associated with yet another class of complex defects. This emission does not appear under optical excitation or via TI, suggesting a distinct excitation pathway. The proposed ML mechanism involves either (i) direct de‐trapping of a hole into an emissive state, or (ii) hole‐mediated transitions through the VB. In this model, state 7 represents a hole trap (HT) located below the VB. Under mechanical force, the trap releases a hole into the VB, which can then be captured by a nearby defect complex (state 8). An electron from a deeper defect level (state 9), located below the Fermi level (E_F_), can subsequently recombine with the trapped hole, yielding the observed green luminescence (transition from level 9 to level 8).^[^ ^39^ ^]^

### 3 Luminescence Thermometry

**Figure 4:**
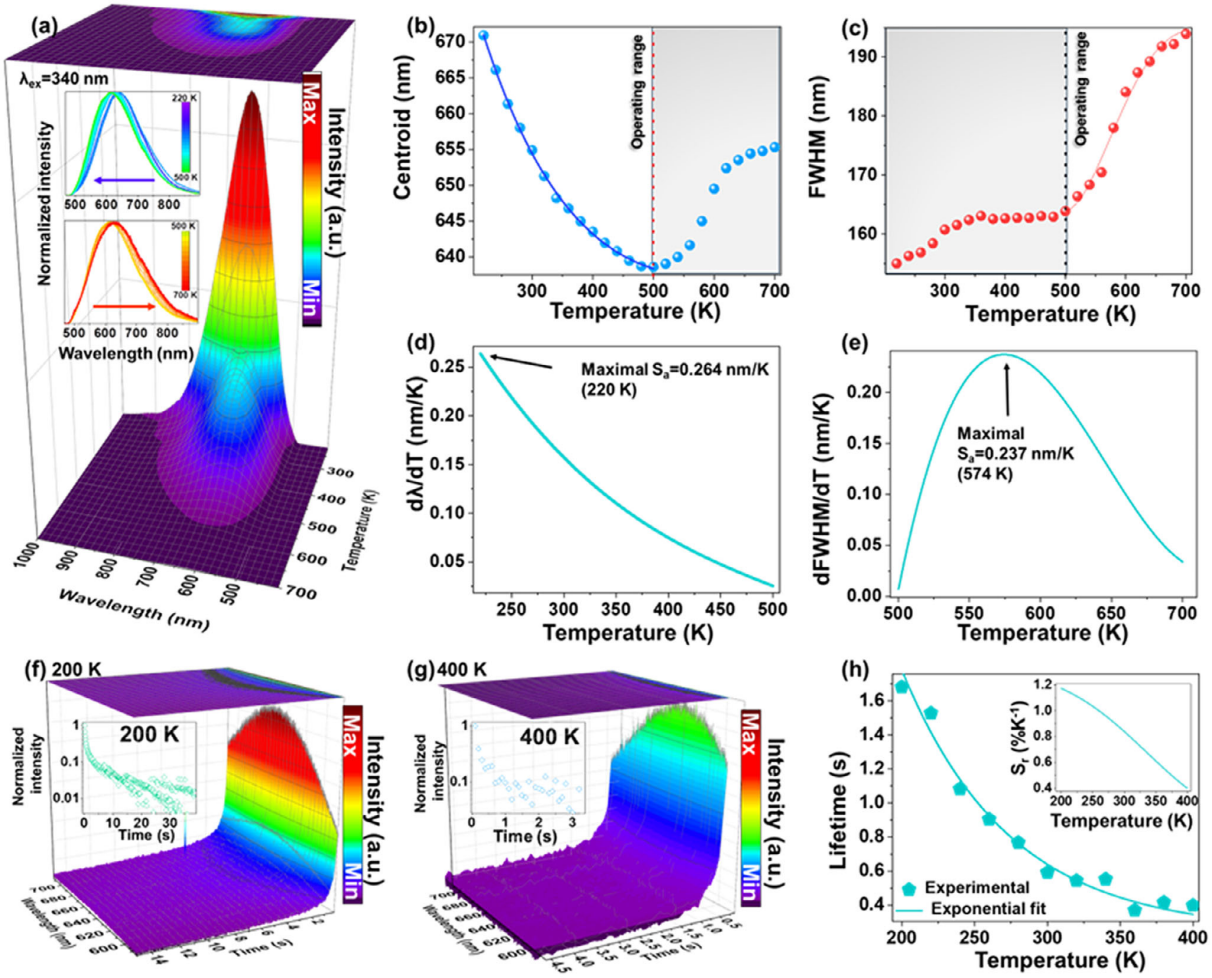
Temperature‐dependent spectroscopic properties of AIN‐4. a) PL spectra of the AlN‐4 material measured at various temperatures (λ_ex_ = 340 nm). Temperature dependence of the emission band b) centroid and c) FWHM. Absolute thermal sensitivities (*S_a_)* determined as d) d*λ*/d*T* for the centroid and e) d*FWHM*/d*T* for the FWHM, plotted as a function of temperature. Time‐resolved PL emission spectra of AlN‐4 measured at f) 200 and g) 400 K, with 0.1 s intervals; insets show the corresponding luminescence decay curves. h) Average luminescence lifetimes determined as a function of temperature; the inset shows the corresponding relative sensitivity (*S_r_)* as a function of temperature.

To explore the thermometric potential of the developed AlN material, its PL emission spectra are systematically measured over a range of temperatures, as presented in **Figure** **4**a. For experimental consistency and due to the availability of a high‐power, focusable light source, the sample was excited at 340 nm, close to the optimal excitation wavelength, producing an intense single‐band emission with a high signal‐to‐noise ratio, suitable for reliable optical sensing applications. As the temperature increased, the PL emission intensity gradually decreased due to thermal quenching effects. Interestingly, the emission band exhibited a spectral shift with temperature. As illustrated in the inset of Figure 4a, the band initially undergoes a blue shift as the temperature increases from 220 to 500 K. Beyond 500 K, the emission band begins to red‐shift with further increase in temperature up to 700 K. According to literature,^[^ ^48^, ^49^ ^]^ AlN remains thermally stable up to 1000 K. The observed spectral evolution is attributed to emission originating from a distribution of defect states with varying energy levels, which respond differently to increasing thermal energy. Thermal quenching mechanisms begin to dominate above 500 K, causing the redistribution of the carrier population among available states and resulting in the observed spectral shift. The temperature dependence of the emission band centroid position and full width at half maximum (FWHM) is presented in Figure 4b,c, respectively. The emission centroid shifts significantly from 671 nm (≈14903 cm^−1^) at 220 K to 638.7 nm (≈15657 cm^−1^) at 500 K, indicating a pronounced blueshift. Above 500 K, the centroid exhibits a monotonic redshift, reaching 661.6 nm at 700 K. This pronounced, monotonic variation in the emission centroid with temperature enhances the sensitivity of the AIN material for precise optical thermometry over a broad temperature range.^[^ ^31^, ^50^ ^]^ Based on these findings, the optimal operational range for using the centroid position as a thermometric parameter is identified as 220–500 K. The thermal evolution of the band centroid fits well to a polynomial function, with an excellent correlation coefficient (*R* ^2^ = 0.999).

(2) $${\lambda \;}_{\mathrm{cent}\mathrm{roid}}\left(\mathrm{or}\;\mathrm{FWHM}\right)=A_{3}T_{3}+A_{2}T_{2}+A_{1}T+A_{0}$$

where *A*_0_, *A*_1_, *A*_2_, and *A*_3_ are the fitting parameters and *T* is the temperature. The corresponding fitting parameters are listed in Table S1 (Supporting Information).

The temperature dependence of the FWHM is shown in Figure 4c. In contrast to the band centroid, the FWHM exhibits a monotonically increasing trend with rising temperature. Notably, the rate of change of the FWHM differs below and above 500 K, corresponding well with the inverse trend observed for the centroid shift in the same temperature intervals. This complementary behavior suggests a transition in the dominant thermal quenching mechanisms ≈500 K. Based on this analysis, the optimal operational temperature range for the AlN‐based optical thermometer, when using FWHM as a sensing parameter, is identified as 500–700 K, where the material demonstrates significantly enhanced sensitivity. Furthermore, the temperature‐dependent FWHM data show an excellent fit to a polynomial function (*R* ^2^ = 0.999), as described by Equation 2. The detailed fitting function and corresponding parameters are provided in the Supporting Information.

The absolute temperature sensitivities (*S*_a_) determined using the band centroid and FWHM as thermometric parameters are presented in Figure 4d,e, respectively. For the band centroid, the absolute sensitivity (defined as d*λ*/d*T)* exhibits a decreasing trend with increasing temperature up to 500 K, reaching a maximum value of 0.264 nm K^−1^ at 220 K. In contrast, the FWHM‐based sensitivity (d*FWHM*/d*T)* reaches its maximum value of 0.237 nm K^−1^ at 574 K. The thermal sensitivities achieved with the AlN‐based sensor surpass those of many previously reported band‐shift luminescent thermometers, particularly those utilizing lanthanide (Ln^3+/2+^) or transition metal ions as luminescence centers. Current state‐of‐the‐art band‐shift thermometers, primarily based on semiconductor nanocrystals and quantum dots, demonstrate sensitivities in the range of 0.100−0.200 nm, such as CdSe(ZnS) (dλ/dT = ≈0.11 nm K^−1^),^[^ ^51^ ^]^ CdTe (dλ/dT = ≈0.193 nm K^−1^),^[^ ^52^ ^]^ and PbSe (dλ/dT = ≈0.16 nm K^−1^).^[^ ^53^ ^]^ In comparison, the AIN‐based thermometer reported here not only exhibits superior sensitivity but also benefits from a wider operational temperature range and the capability for multimodal and multiparameter sensing. As compared to other PL thermometers based on bandshift or bandwidth, i.e., LaF_3_:Nd^3+^ (dλ/dT = ≈0.0068 nm K^−1^), Na_4_Mg(WO_4_)_3_:Mn^4+^ (dλ/dT = 0.127 nm K^−1^),^[^ ^54^ ^]^ YAG:Ce^3+^(dλ/dT = ≈0.047 nm K^−1^),^[^ ^55^ ^]^ SrB_4_O_7_:Tm^2+^ (dFWHM/dT = 0.092 nm K^−1^),^[^ ^56^ ^]^ YAlO_3_: Nd^3+^(dFWHM/dT = ≈0.039 nm K^−1^),^[^ ^57^ ^]^ and Gd_2_ZnTiO_6_: Mn^4+^ (dFWHM/dT = ≈0.090 nm K^−1^),^[^ ^58^ ^]^ the AIN‐based sensor clearly demonstrates absolute superior sensitivity.

The time‐resolved PL spectra of the AlN‐4 sample, recorded in the temperature range of 200–400 K, are shown in Figure S7 (Supporting Information). Two representative series of spectra at the extreme temperatures are shown in Figure 4f,g, respectively. The corresponding decay curves, extracted from the time‐resolved PL data, are shown in Figure S8 (Supporting Information) as well as in the insets of Figure 4f,g. The time‐resolved PL measurements reveal a strong temperature dependence of the luminescence dynamics. Specifically, with increasing temperature, a significant reduction in the luminescence decay time was observed. Given that the recorded luminescence decay curves deviate from a purely exponential behavior, the average excited‐state lifetimes (τ) were calculated to quantitatively assess the thermal evolution of the emission kinetics. The average lifetimes were determined using the following equation:

(3) $$\tau =\frac{\;\int I\left(t\right)\;tdt}{\int I\left(t\right)\;dt}\;$$

where *τ* is the average lifetime and *I* is the emission intensity at time *t*. As the temperature increases from 200 to 400 K, the determined luminescence lifetime of the AlN‐4 material decreases significantly from 1.68 to 0.40 s. The calculated relative sensitivity (S_r_) values, based on the luminescence lifetime as a thermometric parameter, were determined using the following equation:

(4) $$S_{r}=100\%\;\times \left|\left(1/\tau \right)\times \left(d\tau /dT\right)\right|$$

The calculated S_r_ values exhibit a decreasing trend with increasing temperature, with a maximum relative sensitivity of 1.17%/K observed at 200 K, confirming good sensing performance in the kinetic mode, as well.

### 4 Luminescence Manometry and Pressure Stability

**Figure 5:**
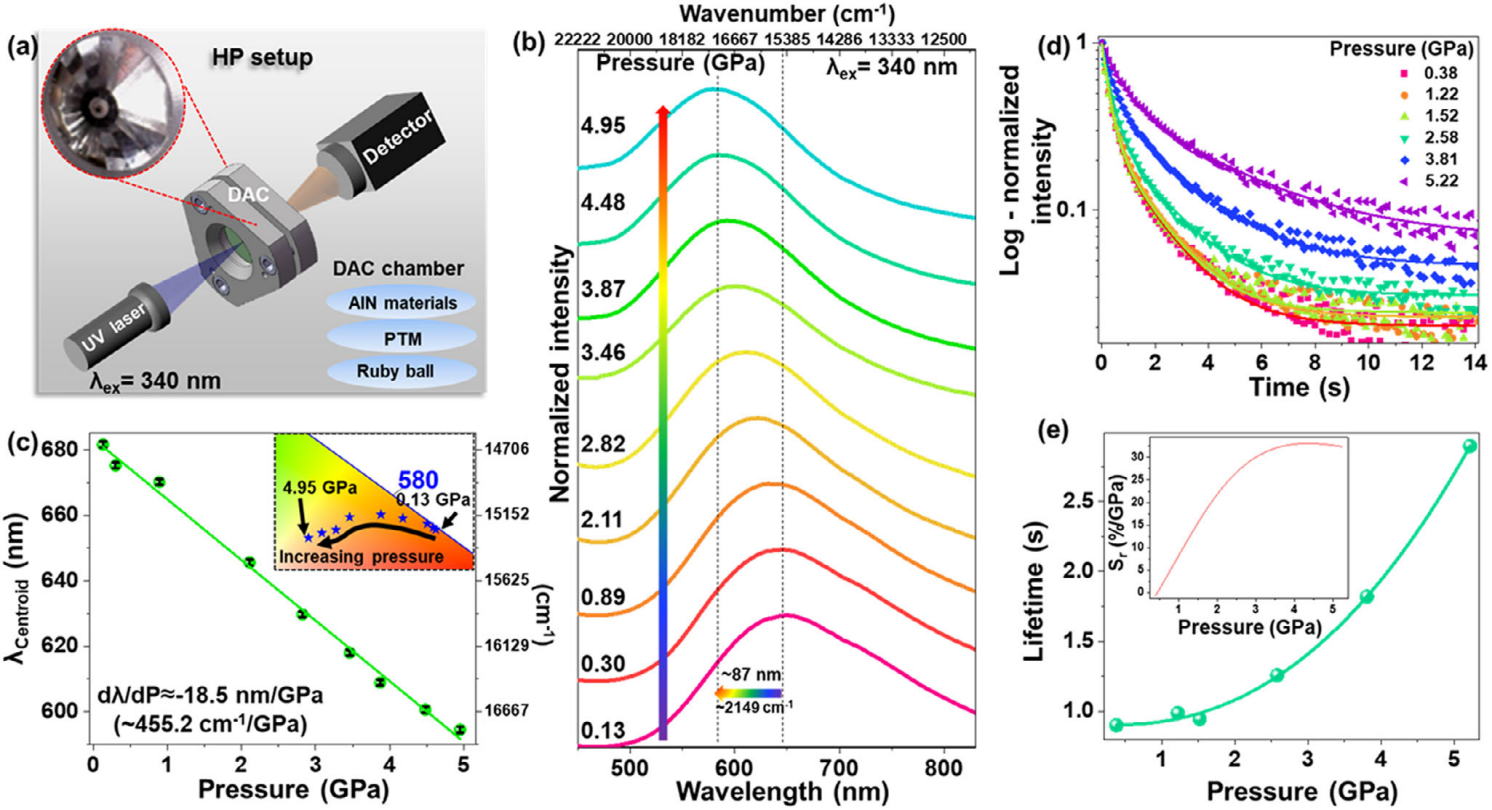
High‐pressure spectroscopic properties. a) Simplified schematic of the HP measurement setup, showing the pressure chamber and the optical geometry used. b) Normalized PL spectra of the AlN‐4 sample at various pressures recorded at 340 nm excitation. c) Emission peak centroids of the AlN as a function of pressure. The inset shows a magnified region of the CIE diagram, indicating the pressure‐induced emission color changes. d) Afterglow, *i.e*., PersL decay curves based on the corresponding time‐resolved emission spectra (from Figure S10, Supporting Information) recorded at selected pressures of 0.38, 1.22, 1.52, 2.58, 3.81, and 5.22 GPa. f) Determined afterglow lifetimes as a function of pressure; the inset shows the corresponding relative pressure sensitivity for sensing in the lifetime‐mode.

To evaluate the effect of pressure on the developed AIN‐4 material and investigate its potential as an optical manometer, PL spectra are measured under varying isostatic pressures using the high‐pressure setup shown in **Figure** **5**a. These measurements were conducted in a diamond anvil cell (DAC), with Al_2_O_3_:Cr^3+^ serving as a pressure indicator. Using a 340‐nm UV lamp as an excitation source, the PL spectra consisted of a single broad emission band, observed consistently up to ≈5 GPa (50 kbar). Notably, the emission band exhibited a linear blue shift with increasing pressure, evidenced by a gradual decrease in the centroid wavelength (Figure 5b).^[^ ^59^, ^60^, ^61^ ^]^ As shown in Figure 5c, the emission centroid shifts from 681.6 nm (14672 cm^−1^) at ambient pressure (0.1 MPa) to 594.5 nm (16821 cm^−1^) at 4.95 GPa, resulting in a high and constant pressure sensitivity of 18.5 nm GPa^−1^ across the full examined range. The corresponding pressure‐dependent chromaticity coordinates were plotted on the Commission Internationale de l’Éclairage (CIE) diagram (Figure S9, Supporting Information), and the corresponding CIE coordinates are shown in Table S2 (Supporting Information). The inset in Figure 5c shows a magnified region of the CIE diagram, depicting a significant color change from red to yellow with increasing pressure, thus indicating the capability of the AIN‐4 material as a visual pressure sensor. Additionally, time‐resolved PL spectra are recorded at different pressures (0.38, 1.22, 1.52, 2.58, 3.82, and 5.22 GPa), as shown in Figure S10 (Supporting Information), with the corresponding afterglow decay curves presented in Figure 5d. The results indicate that pressure significantly influences the luminescence kinetics. Figure 5e depicts the average afterglow lifetimes determined as a function of pressure. Since the decay curves deviated from a purely exponential nature, the average lifetimes were calculated to quantify the pressure‐dependent evolution of emission kinetics, following the methodology described for the temperature‐dependent studies.

Interestingly, the AlN material exhibits a rarely reported phenomenon, a significant prolongation of afterglow lifetimes under high‐pressure conditions. Specifically, the luminescence lifetime increases from ≈1 s to almost 3 s at ≈5 GPa. This effect is plausibly associated with increased depth of defect states and trap states within the compressed structure. The evolution of the average lifetimes as a function of pressure is also described by a polynomial function, with the corresponding fitting parameters listed in Table S1 (Supporting Information). The unique behavior enables the use of the AlN material as a pressure sensor in time‐resolved mode, functioning as a super‐sensitive optical manometer based on the PersL lifetime. Notably, the material achieves a remarkable relative sensitivity of 33.1%/GPa.

Overall, the developed AIN‐based sensor exhibits two distinct and complementary pressure sensing modes—lifetime‐based and band shift modes, with remarkably high sensitivities of 18.5 nm GPa^−1^ (455.2 cm^−1^/GPa) and 33.1%/GPa, respectively. In addition to its high‐pressure sensitivity, the material displays excellent thermal and chemical stability. Notably, the band‐shift sensitivity of this sensor is over 50 times greater than that of the widely used ruby pressure standard (dλ/dp = 0.365 nm GPa^−1^),^[^ ^29^ ^]^ and ≈75 times higher than that of SrB_4_O_7_:Sm^2+^ (dλ/dP ≈0.25 nm GPa^−1^).^[^ ^30^, ^62^, ^63^ ^]^ Furthermore, its performance significantly surpasses that of most previously reported sensors.^[^ ^46^, ^58^, ^59^, ^60^, ^64^, ^65^, ^66^, ^67^ ^]^ Although some recently developed pressure sensors exhibit even higher sensitivities, for example, the Bi^3+^‐doped double perovskite Cs_2_Ag_0.6_Na_0.4_InCl_6_ manometer developed by our group achieved an extremely high sensitivity of ≈112 nm GPa^−1^. However, it operates only up to 4 GPa. Moreover, these materials suffer from structural instability, photo bleaching, chemical instability, and humidity sensitivity, limiting their practical applications. Moreover, their extremely broad emission bands (e.g., FWHM = 235 nm) reduce the precision of pressure measurements. Similarly, although Mn^2+^‐activated Zn_2_GeO_4_ phosphor can reach a sensitivity of 21.3 nm GPa^−1^, this is only achieved at pressures above 6.76 GPa, while their low‐pressure sensitivity remains limited (≈8 nm GPa^−1^). This study indicates, for the first time, that defect‐related emissions in undoped AIN can exhibit strong and highly tunable pressure sensitivity, providing a powerful platform for optical pressure and temperature sensing. The exceptional sensitivity, combined with high durability and the ability to enable visual pressure sensors (e.g., via simple UV light sources and direct observation), makes this material especially promising for applications in infrastructure monitoring, quality inspection, and disaster prevention in building collapse, where low‐cost, passive, and reliable sensors are crucial.

**Figure 6:**
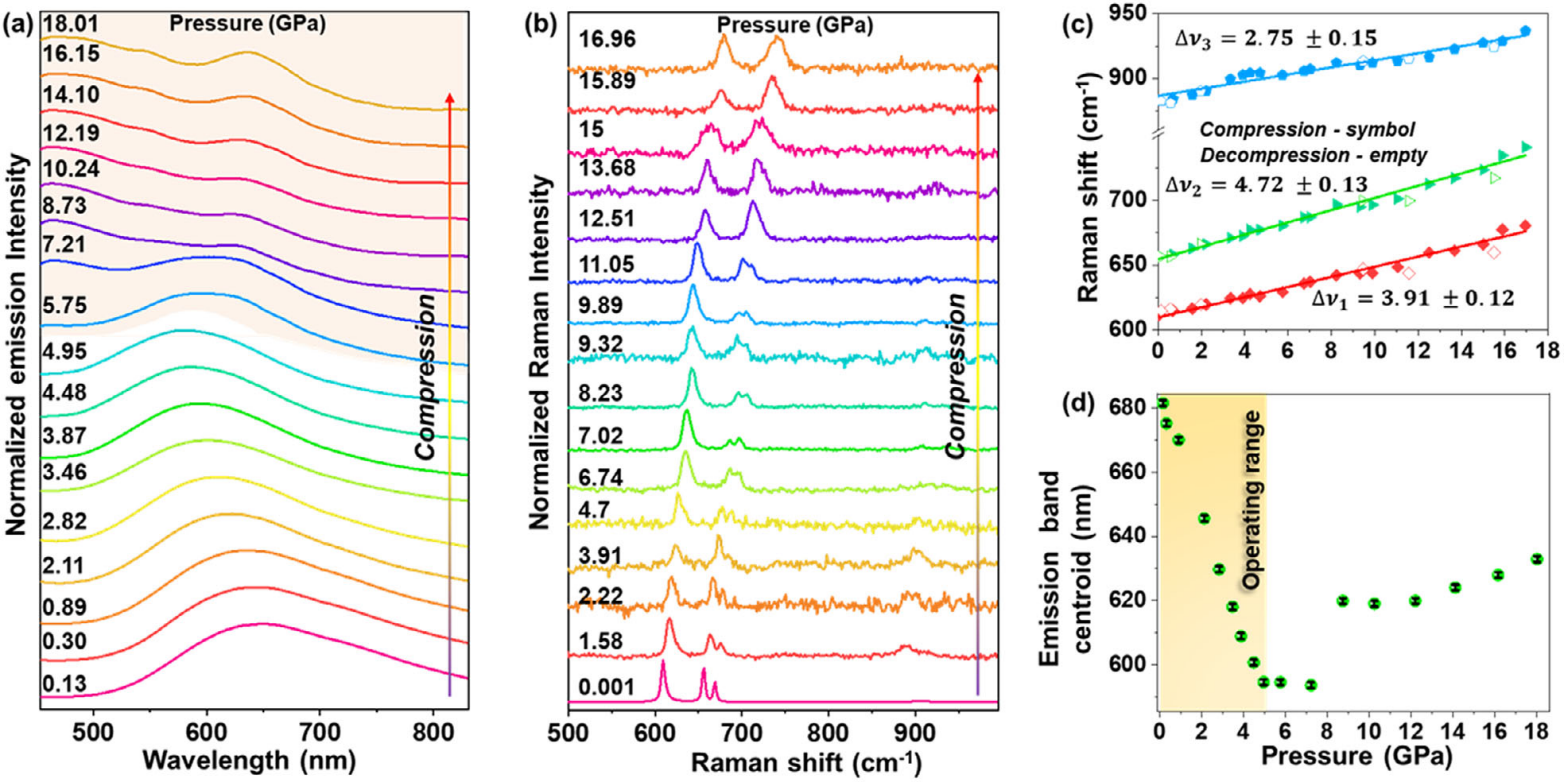
High‐pressure stability performance. a) Normalized emission spectra of the AlN‐4 material, recorded at increasing pressures, using 340 nm excitation. b) High‐pressure Raman spectra under compression. c) Corresponding Raman mode energies and d) emission peak centroid for the AlN‐4 sample as a function of pressure; the continuous lines represent the performed linear fits, filled symbols represent the data in compression, and empty symbols represent that in decompression.

To investigate the pressure stability and detailed spectroscopic characteristics of the developed AlN sensor material, systematic PL measurements were performed under high‐pressure conditions up to ≈18 GPa. Raman spectroscopy was employed on the compressed material (up to ≈17 GPa) to probe its structural and vibrational properties under extreme conditions. This combined spectroscopic approach enables a direct correlation between the pressure‐dependent optical emission characteristics and the lattice dynamics, providing comprehensive insights into the structural integrity and phase stability of the material. As shown in **Figure** **6**a, when the applied pressure exceeds 5–6 GPa, the PL emission behavior deviates from the previously observed linear blue shift. The emission band progressively becomes asymmetric and exhibits a redshift.

To further investigate the influence of applied pressure on the AlN structure, Raman spectroscopy was systematically performed as a function of pressure. At ambient conditions, the Raman spectrum of the AlN‐4 sample exhibits several well‐defined bands corresponding to Raman‐active phonon modes, with the most intense peaks centered at ≈611.4, 656.5, and 884.0 cm^−1^ (Figure 6b). Upon increasing the pressure up to 16.96 GPa, all observed phonon modes exhibit a gradual, linear shift toward higher energy values (higher wavenumbers), consistent with phonon hardening due to lattice compression. The Raman bands show a constant shift rate for the mentioned bands of 3.91, 4.72, and 2.75 cm^−1^/GPa, respectively (Figure 6c), indicating excellent pressure stability of the developed sensor. This pressure‐induced shift in Raman frequencies is attributed to the reduction Al─N bonds, as reduced interionic distances enhance bond stiffness under compression. Importantly, no abrupt changes in the Raman mode energies or appearance of new vibrational features were observed throughout the pressure range studied, indicating the absence of pressure‐induced phase transitions up to ≈17 GPa. These results are in good agreement with previous reports, which suggest that AIN undergoes phase transitions only at pressures exceeding ≈20 GPa.

In contrast, the PL band centroid (Figure 6d), originating from the defect‐related emission in the AlN crystal, exhibits a linear blue shift only up to ≈5 GPa. Beyond this pressure, the band centroid deviates notably from this monotonic trend, with the shift rate slowing and even reversing in some cases. This change is plausibly attributed to pressure‐induced variation of the defect state types and energies, and modifications in the local symmetry of the AlN crystal lattice above 5 GPa. These structural and electronic perturbations disrupt the initially stable configuration responsible for the blue‐shifted emission observed from 0.1 MPa to 5 GPa. In addition, pressure‐induced changes in the defect states or carrier localization effects may contribute to the observed asymmetry and redshift in the emission band, reflecting altered charge carrier dynamics under extreme compression. Importantly, such local rearrangements can also shift defect or trap levels relative to the host band edges more strongly than the bandgap itself, thereby modifying carrier capture and recombination pathways. As pressure continues to increase, these altered energy alignments together with enhanced electron–phonon interactions can lower activation barriers for nonradiative decay, accelerate multiphonon relaxation, or even enable resonances between trap states and bands, ultimately accounting for the observed quenching and spectral distortion beyond 5 GPa.

Noteworthy, the slower response times associated with defect‐related emission in the developed sensor, when compared to conventional luminescence sensors, may present a potential drawback, particularly for applications requiring high‐speed sensing. In addition, the long‐term stability and durability of defect‐induced luminescence under repeated mechanical or optical excitation remain concerns that could hinder practical deployment in harsh or dynamic environments. Several other factors should be considered for such a sensor. For instance, the defect density and distribution may be important. Thus, careful control of the defect creation process (e.g., through annealing or heat‐tr) will be crucial for ensuring the sensor accuracy and sensitivity. Additionally, factors such as chemical stability, response time, and sensitivity to stress, temperature, and pressure should be considered, as all are crucial for the practical use of such optical sensors. Nevertheless, the excellent ML properties of AlN make it a highly promising material for the development of self‐powered stress and strain sensors for structural health monitoring in aerospace, civil engineering, and robotics. Furthermore, these characteristics position AlN as an attractive candidate for next‐generation smart devices capable of integrating real‐time mechanical stress sensing with luminescent temperature and pressure sensing, offering excellent sensitivity and multifunctional performance.

## 2 Conclusion

Here, we report the first example of a defect‐based optical pressure and temperature sensor based on undoped AlN. Considering the combination of PL, afterglow emission, and ML properties, undoped AlN emerges as a truly multifunctional sensing material, capable of simultaneous force, pressure, and temperature detection. A series of piezoelectric AlN samples was synthesized under various conditions and systematically optimized for enhanced opto‐mechanical performance. The optimized AIN‐4 composition demonstrated remarkable improvements of up to 1–2 orders of magnitude in sensing performance compared to conventional luminescent sensor materials. Currently, we have observed previously unreported spectral variations in PL, afterglow, and ML emissions under different excitation modes (optical and mechanical), attributed to the involvement of distinct defect states in the emission processes. The developed AlN‐based multifunctional sensing platform exhibits extraordinary pressure and temperature sensitivities, making it a highly promising candidate for next‐generation optical sensing and signal transmission technologies with potential applications spanning telecommunication, engineering, and space technologies.

## 3 Experimental Section

Detailed experimental procedures are reported in the Supporting Information.

## Conflict of Interest

The authors declare no conflict of interest.

## Supporting information



Supporting Information

Supplemental Video 1

## Data Availability

The data that support the findings of this study are available from the corresponding author upon reasonable request.
