# Supplementary material for: Mechanoluminescent Aluminum Nitride Crystal for Super‐Sensitive Optical Manometry, Thermometry and Force Sensing
Source: Adv Mater. 2025 Sep 9;38(1):e11943. doi: 10.1002/adma.202511943 (PMC12759223; doi:10.1002/adma.202511943)
Supplement: Supplementary file 1 — Supporting Information [file ADMA-38-e11943-s001.docx]

**Supporting Information for**

**Mechanoluminescent Aluminum Nitride Crystal for Super-sensitive Optical Manometry, Thermometry and Force Sensing**

*Teng Zheng,* *Przemysław Woźny, Kevin Soler-Carracedo, Dongxue Han, Jie Wang, Liang Peng, Wenliang Li, Dengfeng Peng,* Honglei Wu,* Jan Moszczyński, Sebastian* *Mahlik, Marcin Runowski**

Dr. T. Zheng, Dr. Dongxue Han, Dr. Jie Wang, Prof. Liang Peng

School of Information and Electrical Engineering,

Hangzhou City University

Hangzhou 310015, China

Dr. P. Woźny, Dr. K. Soler-Carracedo, J. Moszczyński, Prof. M. Runowski,

Faculty of Chemistry

Adam Mickiewicz University

Poznań 61-614, Poland

E-mail: runowski@amu.edu.pl

Prof. S. Mahlik,

Institute of Experimental Physics

Faculty of Mathematics, Physics and Informatics,

Wita Stwosza 57

University of Gdansk,

Gdansk 80-308, Poland

Prof. D. Peng

Shenzhen Key Laboratory of Intelligent Optical Measurement and Detection, Shenzhen University Shenzhen 518060, China; Key Laboratory of Optoelectronic Devices and Systems of Ministry of Education and Guangdong Province, College of Physics and Optoelectronic Engineering, Shenzhen University, Shenzhen 518060, China; State Key Laboratory of Radio Frequency Heterogeneous Integration, Shenzhen University. Shenzhen 518060, China

E-mail: [pengdengfeng@szu.edu.cn](mailto:pengdengfeng@szu.edu.cn)

W. Li, Prof. H. Wu

Key Laboratory of Optoelectronic Devices and Systems of Ministry of Education and Guangdong Province, College of Physics and Optoelectronic Engineering, Shenzhen University, Shenzhen 518060, China; State Key Laboratory of Radio Frequency Heterogeneous Integration, Shenzhen University, Shenzhen 518060, China

E-mail: hlwu@szu.edu.cn

**Keywords:** Optical manometry; luminescent temperature sensor; mechanoluminescence; aluminium nitride; ultra-wide bandgap semiconductor.

**Methods**

**Synthesis and Materials**

A metal tungsten resistance heating system was used to perform the high-temperature sintering under a nitrogen atmosphere and purification of AlN powder, within a pressure range of 0.7 atm to 1 atm and a temperature range of 2000 °C to 2300 °C. During the experiment, the effects of different temperature conditions on the purification of commercially available AlN powder were systematically investigated by controlling the sintering temperature and time. After the sintering and purification process, the treated AlN powder was classified by sintering conditions, labeled as AlN-1, AlN-2, AlN-3 and AlN-4, *i.e*. 2000 ℃ for 60 hours (named AlN-1), 2150 ℃ for 60 hours (AlN-2), 2300 ℃ for 60 hours (AlN-3) and 2300 ℃ for 100 hours (AlN-4).

**Structural and Morphological Characterization.** X-ray diffraction (XRD) analysis was conducted on a Bruker D8 Advance diffractometer equipped with a Cu Kα1 radiation source (λ = 1.5406 Å). Data were collected in the angular range of 10°–60° with a step size of 0.05°. Morphological features and elemental distribution were analyzed using a field emission scanning electron microscope (FEI Quanta 250 FEG) coupled with an EDAX energy-dispersive X-ray spectrometer. Backscattered electron imaging and energy-dispersive spectroscopy (EDS) were performed to correlate structural and compositional properties.

**Ambient Photoluminescence Analysis.** Steady-state photoluminescence (PL) spectra under ambient conditions were acquired using an Andor Shamrock 500i spectrograph paired with an iDus silicon CCD detector. Powder samples were dispersed in a quartz sample holder, and emission-excitation spectra were recorded following system response correction to ensure spectral accuracy.

**High-Pressure DAC Setup.** High-pressure experiments were carried out utilizing a Merrill-Bassett-type diamond anvil cell (DAC). Pressure regulation was achieved via three stainless-steel screws, with a pre-indented stainless-steel gasket (thickness:50 μm; initial aperture: ~150 μm) defining the sample chamber. A methanol/ethanol/water (16:3:1, vol.) mixture served as the hydrostatic pressure-transmitting medium. A micron-sized ruby chip (<10 μm) was co-loaded with the sample for in situ pressure monitoring. PL properties under compression were investigated using a 300 nm UV diode as the excitation source, with backscattered geometry for emission collection. Emission spectra were recorded at room temperature, and intensity corrections were applied to account for instrument response variations. Pressure values within the DAC were determined via the ruby fluorescence R_1_ line shift, calibrated according to the empirical standard curve provided by *Mao H.K., Xu J., and Bell P.M. (1986) J. Geophys. Res. 91, 4673*. Raman spectra under pressure were recorded in a backscattering geometry using a confocal micro-Raman system (Renishaw InVia), equipped with a grating of 1800 grooves per mm and a power-adjustable 532 nm laser diode. All spectra were corrected for the system's apparatus response.

**Temperature dependent measurement.** Fluorescence excitation and emission spectra of the designed AlN materials were recorded using a fluorescence spectrometer (Edinburgh FS5) equipped with a temperature control system (Linkam HFS600E-PB2). Additionally, the emission decay curves of the AlN materials were measured using a fluorescence spectrometer (Edinburgh FS1000).

**ML measurement.** For the ML measurement, a film bonded with the AlN material was prepared using EVA-PET sealing plastic film. The ML spectra emitted at various force levels were then measured using a custom-built ML testing platform, which included a stepper motor, a digital push-pull force gauge, and an Ocean Optics QE65pro fiber optic spectrometer. During the measurements, the ML material was fixed onto an organic glass substrate using double-sided tape. A metal tip, connected to the digital push-pull force gauge, was used to apply a fixed pressure to the film. The stepper motor was employed to drive the force gauge in a lateral motion. A fiber optic spectrometer, positioned on the opposite side of the organic glass, was used to collect the emitted light during the sliding motion and to perform subsequent calculations and measurements.


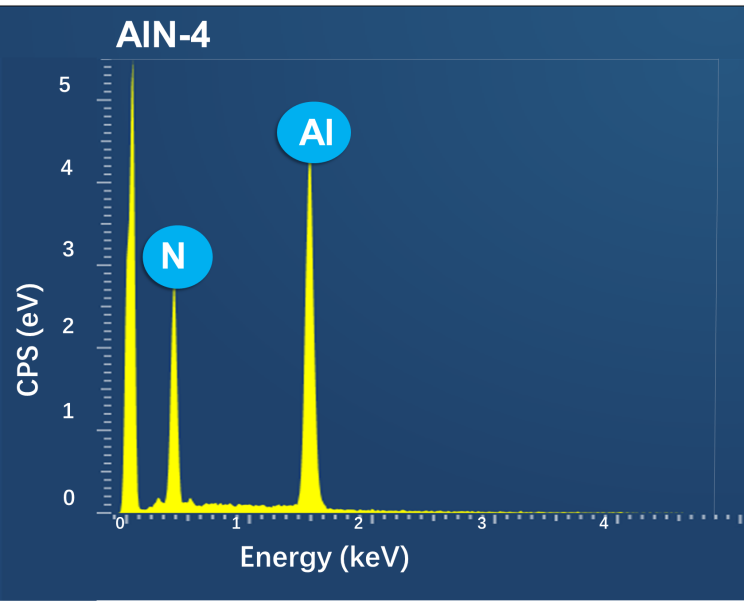


**Figure S1**. EDX spectrum of the AlN-4 sample.


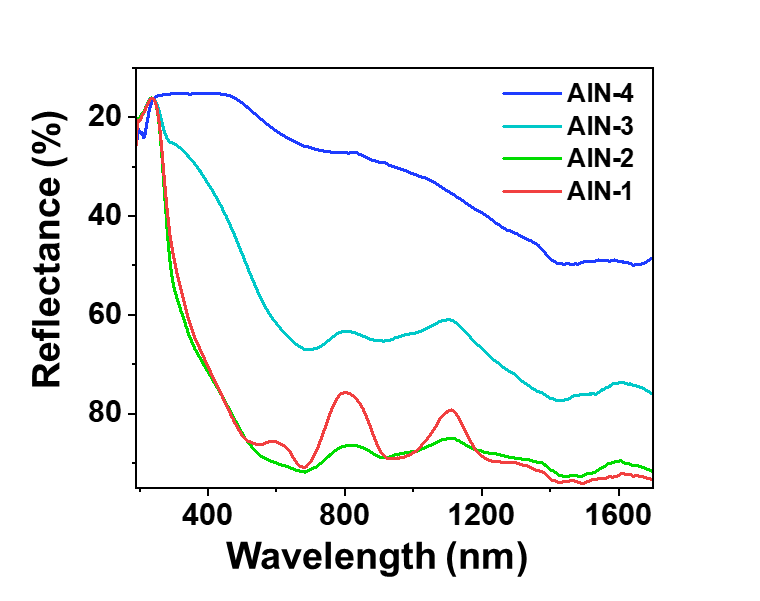


**Figure S2**. (a) Reflectance spectra for the materials AlN-1, AlN-2, AlN-3 and AlN-4.


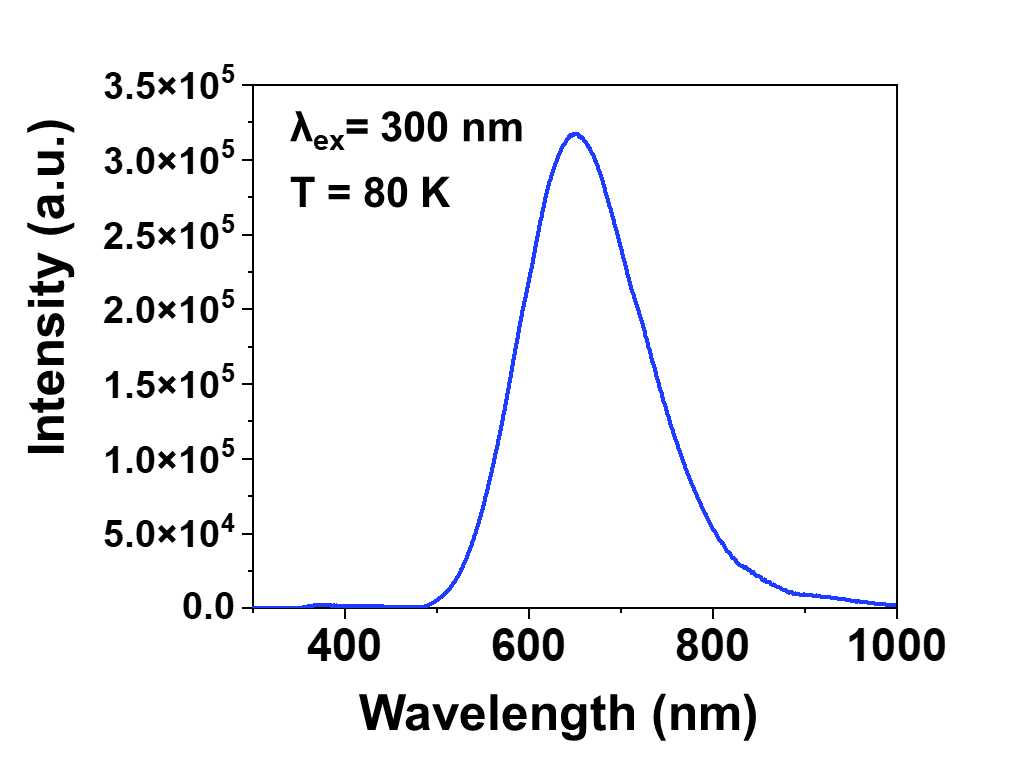


**Figure S3**. The PL spectrum of the AlN-4 sample measured at 80 K.


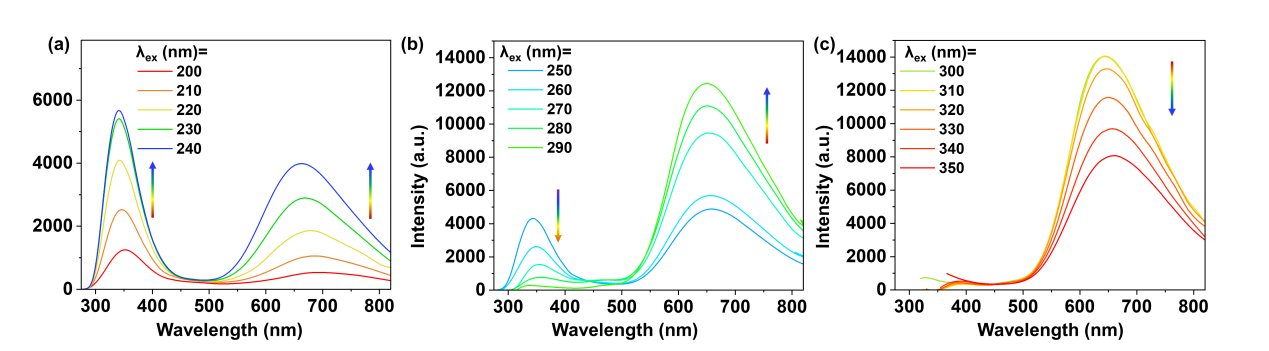


**Figure S4.** The non-normalized PL emission spectra of the AlN-4 sample as a function of λ_ex_. (a) λ_ex_ = 200-240 nm, both the UV band and red band shows increasing tendency, (b) λ_ex_ = 250-290 nm, UV band decreases and red band increases. (c) λ_ex_ = 300-350 nm, UV band decreases to noise level and red band decreases.


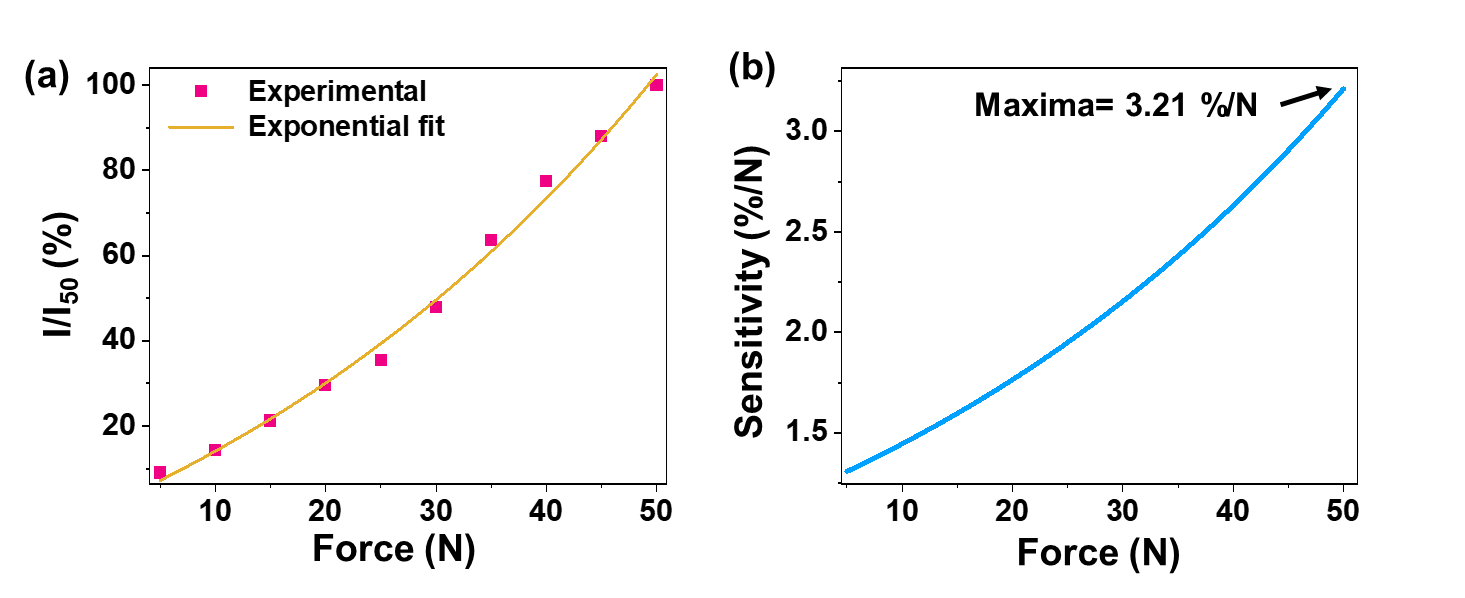


**Figure S5**. (a) The dependence of *I/I_50_* value on applied force for the AIN-4 sample, with experimental data fitted to an exponential function. *I/I_50_* represents the relative intensity at different forces, compared to ML intensity at F = 50 N (*I_50_*). (b) The corresponding sensitivity in % as a function of applied force.


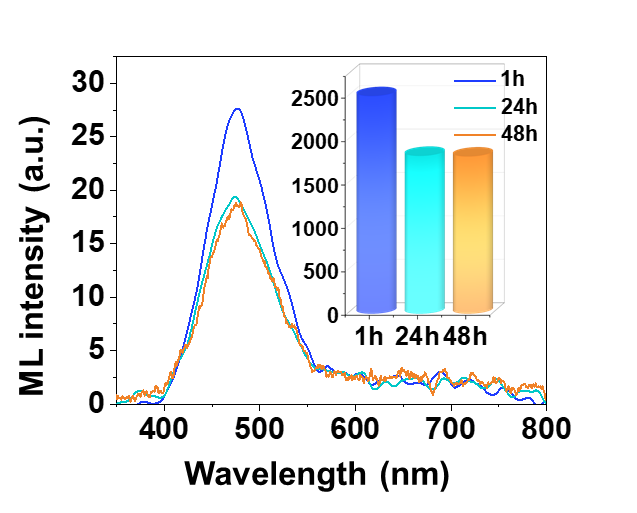


**Figure S6**. ML spectra measured 1, 24, and 48 h after UV irradiation.


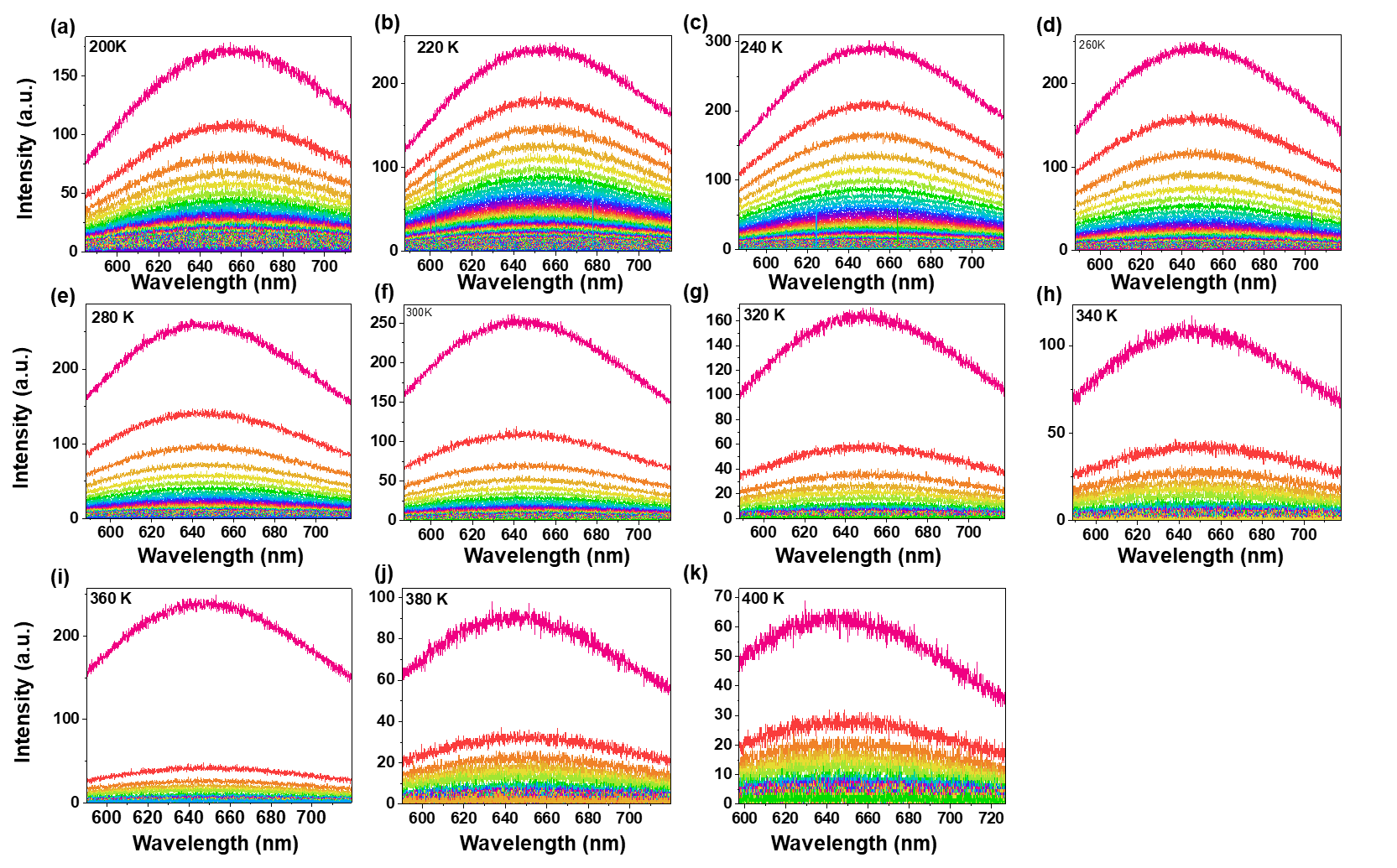


**Figure S7.** Time-resolved photoluminescence spectra recorded for the AlN-4 material at varied temperatures, ranging from 200 to 400 K, with 0.1s interval.


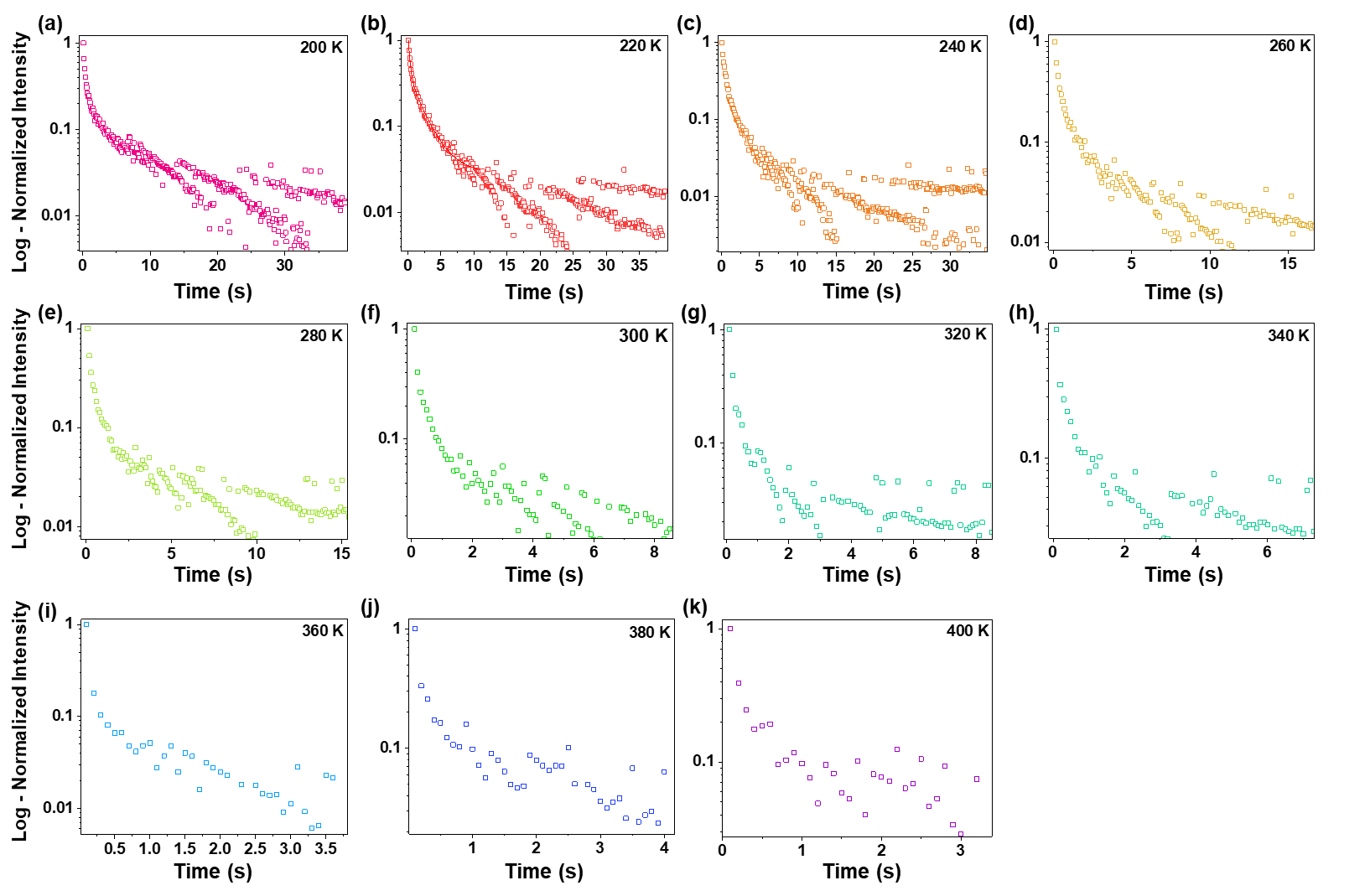


**Figure S8.** The corresponding luminescence decay curves, determined based on the time-resolved emission spectra from Figure S7.


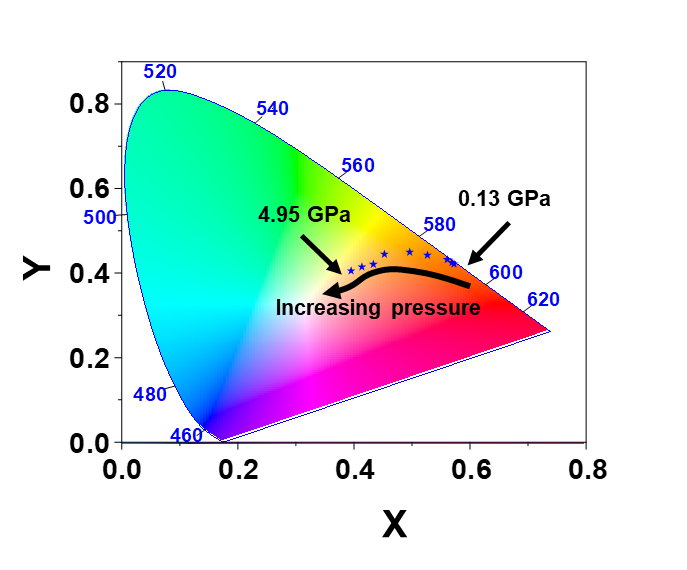


**Figure S9.**The Commission Internationale de l’Éclairage (CIE) chromaticity diagram, showing the PL emission color at various pressure for the developed AlN pressure sensor.


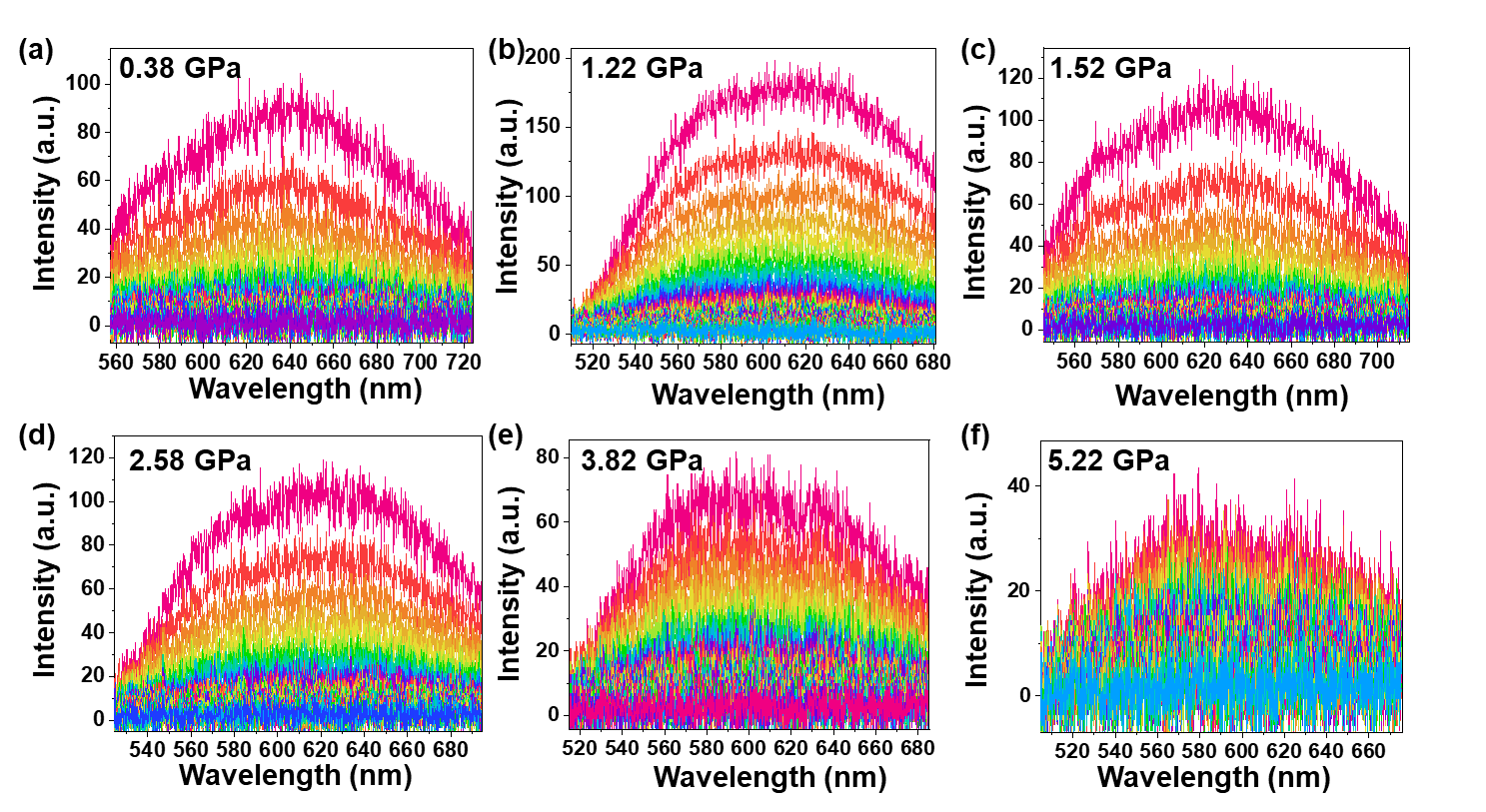


**Figure S10.** Time-resolved emission (afterglow) spectra recorded for the AlN-4 material at different pressures; 0.1 s interval.

**Table S1.** Fitting functions and the related parameters.

|  | ***Fitting function*** | ***Fitting parameters*** | | | | | ***R^2^*** |
| --- | --- | --- | --- | --- | --- | --- | --- |
| Total ML vs. applied Force | Intensity = A_2_exp(-x/t_2_)+ A_1_exp(-x/t_1_)+ A_0_ | *A_2_*=10.18 | *A_1_*=9.70 | *A_0_*=-18.94 | *t_2_*=-45.58 | *t_1_*=-45.57 | 0.999 |
| Emission band centroid vs. Temperature | *λ_centroid_* = *A*_4_*T*^4^ + *A*_3_*T*^3^ + *A*_2_*T*^2^ + *A*_1_*T* + *A*_0_ | *A_4_*=-1.07×10^-9^ | *A_3_*=-2.06×10^-6^ | *A_2_*=0.002 | *A_1_*=-0.86 | A_0_=784.99 | 0.999 |
| Emission band FWHM vs. Temperature | *FWHM* = *A*_4_*T*^4^ + *A*_3_*T*^3^ + *A*_2_*T*^2^ + *A*_1_*T* + *A*_0_ | *A_4_*=-1.144×10^-8^ | *A_3_*=-1.58×10^-5^ | *A_2_*=0.0078 | *A_1_*=-1.58 | A_0_=266.68 | 0.999 |
| Emission lifetime vs. Temperature | *Lifetime* = *A*_2_×exp*(-T/A_1_)* + *A*_0_ | *A*_2_=23.32 | *A*_1_=73.53 | *A*_0_=0.246 |  |  | 0.91 |
| Emission lifetime vs. Pressure | *Lifetime = A_3_P^3^+A_2_P^2^+A_1_P+A_0_* | *A_3_*=0.0046 | *A_2_*=0.059 | *A_1_*=-0.054 | *A_0_*=0.917 | - | 0.997 |

**Table S2**. The (x, y) coordinates of the CIE diagram at various pressure conditions.

| ***Pressure (GPa)*** | ***x*** | ***y*** |
| --- | --- | --- |
| 0.13 | 0.57253 | 0.4214 |
| 0.3 | 0.56888 | 0.42482 |
| 0.89 | 0.56082 | 0.43195 |
| 2.11 | 0.52655 | 0.44248 |
| 2.82 | 0.49609 | 0.4496 |
| 3.46 | 0.45252 | 0.44469 |
| 3.87 | 0.4335 | 0.42065 |
| 4.48 | 0.41385 | 0.41456 |
| 4.95 | 0.39509 | 0.40518 |
